# Supplementary material for: Variable food alters responses of larval crown-of-thorns starfish to ocean warming but not acidification
Source: Commun Biol. 2023 Jun 14;6:639. doi: 10.1038/s42003-023-05028-1 (PMC10267210; doi:10.1038/s42003-023-05028-1)
Supplement: Supplementary file 2 — Supplementary Information [file 42003_2023_5028_MOESM2_ESM.pdf]

Supplementary Information

Supplementary for: Variable food alters responses of larval crown-of-thorns starfish to ocean warming but not acidification

Benjamin Mos[1,2], Naomi Mesic[3], Symon A. Dworjanyn[3]

[1] Moreton Bay Research Station (MBRS), School of Biological Sciences, The University of Queensland, 37 Fraser Street, Dunwich, Minjerribah, QLD 4183, Australia

[2] Centre for Marine Science (CMS), The University of Queensland, Brisbane, QLD 4072, Australia

[3] National Marine Science Centre, Faculty of Science and Engineering, Southern Cross University, PO Box 4321, Coffs Harbour, NSW 2450, Australia

Corresponding Author: B Mos

Email: b.mos@uq.edu.au

Figures: 2

Tables: 3

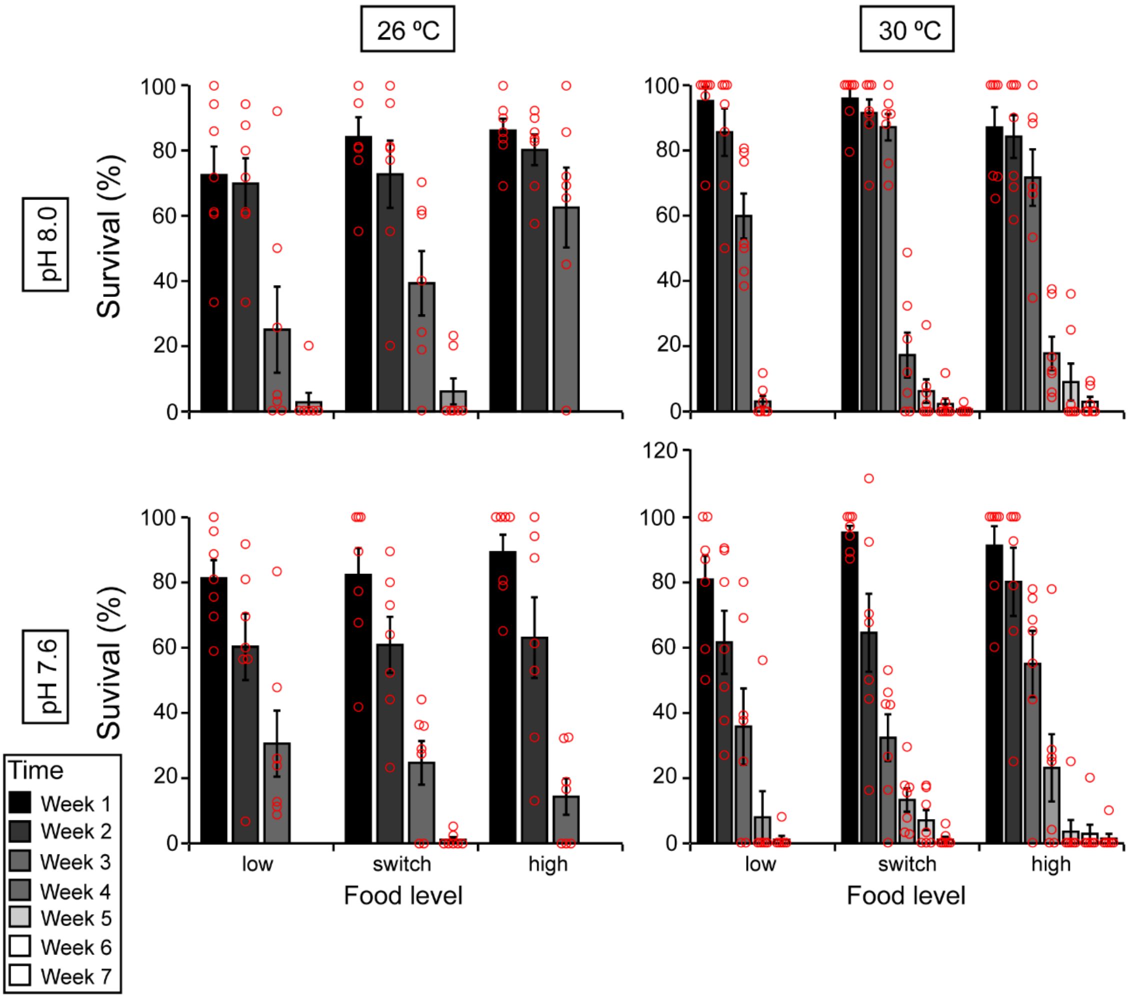

Supplementary Figure 1. Survival (%) of *Acanthaster* sp. larvae reared at two temperatures and two pH levels in a flow-through seawater system. Larvae were fed *Proteomonas sulcata* three times per day at a rate equivalent to 1×10^3 cells mL^{-1} (low), 1×10^3 cells mL^{-1} from 3–11 dpf and 5×10^4 cells mL^{-1} thereafter (switch), or 5×10^4 cells mL^{-1} (high). Survival was followed in each replicate until >50% of living larvae in the replicate became competent to settle or all larvae died (up to 8 weeks post-fertilisation). Survival was calculated using data collected for all living larvae regardless of their morphology (i.e. including abnormal and normal larvae). Red circles represent replicates. Bars represent means ± SE, *n* = 7.

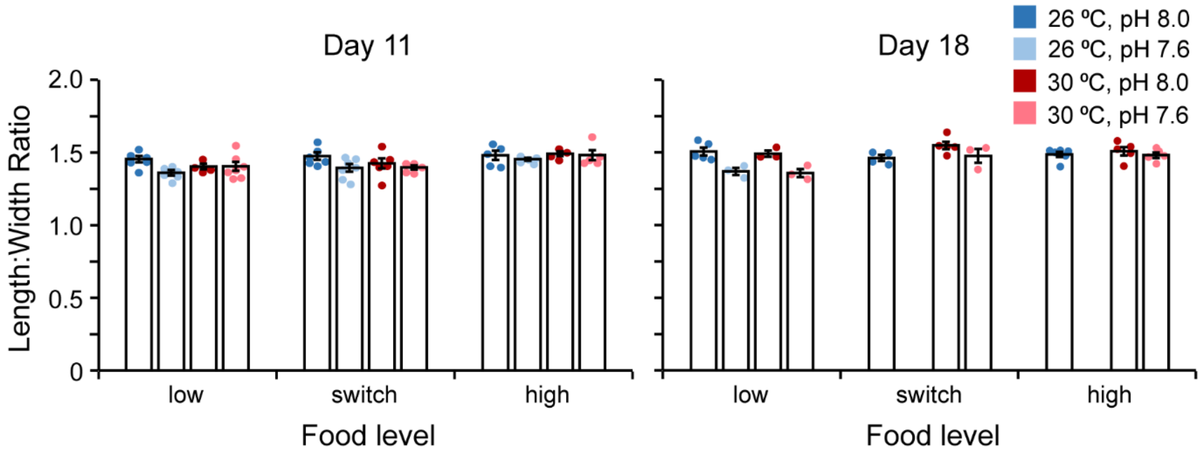

Supplementary Figure 2. The length:width ratio of *Acanthaster* sp. larvae reared at two temperatures (26, 30 °C), two pH levels (pH 8.0, 7.6), and three food level treatments (low, switch, high) in a flow-through seawater system. Larvae were fed *Proteomonas sulcata* three times per day at a rate equivalent to 1×10^3 cells mL^{-1} (low), 1×10^3 cells mL^{-1} from 3–11 days post-fertilisation and 5×10^4 cells mL^{-1} thereafter (switch), or 5×10^4 cells mL^{-1} (high). Bars represent means. Error bars are standard errors of the mean. Data for the 26-7.6-switch and 26-7.6-high treatments at 18 dpf were not presented due to low replication ($n < 3$) because of high mortality (Supplementary Figure S1) and a high occurrence of larvae with abnormal morphology in these treatments. The length:width ratio of larvae at day 11 was influenced by the pH of their culture water (pH 8.0 > 7.6) and the amount of microalgae they were fed (low = switch < high), but not by temperature or any interaction among these factors (Supplementary Table 3). The length:width ratio of larvae at day 18 was influenced by the pH of their culture water (pH 8.0 > 7.6), but not by temperature, food level, or any interaction among these factors (Supplementary Table 3).

Supplementary Table 1. Outcomes of ANOVA analyses examining the effects of two temperatures (26, 30 °C), two pH levels (pH 8.0, 7.6), and three food treatments (low, switch, high) on the length, width, % abnormality, survival (week 1–3), and development to early brachiolaria or late brachiolaria of *Acanthaster* sp. larvae at 11 or 18 dpf (days post-fertilisation) respectively in a flow-through seawater system. Larvae were fed *Proteomonas sulcata* three times per day at a rate equivalent to 1×10^3 cells mL^{-1} (low), 1×10^3 cells mL^{-1} from 3–11 dpf and 5×10^4 cells mL^{-1} thereafter (switch), or 5×10^4 cells mL^{-1} (high). df, degrees of freedom; MS, mean square; temp, temperature; rep., replicate. Significant factors are in bold (*p* < .05).

Parameters	Source	df	MS	F	*p*	Post hoc tests	
Survival	**temp**	**1**	**1.28E4**	**5.32**	**.0238**		
week 1–3	pH	1	1.18E4	2.81	.096		
	food	2	1.72E3	1.99	.142		
	**week**	**2**	**3.83E4**	**137.68**	**.0001**		
	temp × pH	1	1.11E3	1.48	.228		
	temp × food	2	136.19	0.42	.648		
	**temp × week**	2	**1.59E3**	**5.73**	**.0049**	*Interaction*	*Interaction*
	pH × food	2	416.29	0.48	.617	week 1,3 (26 < 30)	26 (week 1 > 2 > 3)
	**pH × week**	**2**	**3.44E3**	**12.38**	**.0001**	week 2 (26 = 30)	30 (week 1 > 2 > 3)
	food × week	4	148.93	0.54	.708		
	temp × pH × food	2	2.22E3	1.71	.154	*Interaction*	*Interaction*
	temp × pH × week	2	77.96	0.28	.755	week 1 (8.0 = 7.6)	8.0 (week 1 > 2 > 3)
	temp × food × week	4	116.44	0.42	.798	week 2,3 (8.0 > 7.6)	7.6 (week 1 > 2 > 3)
	pH × food × week	4	591.74	2.13	.086		
	temp × pH × food × week	4	597.61	2.15	.074		
	**rep. (temp × pH × food)**	**72**	**858.50**	**3.09**	**.0001**		
	residual	144	277.91				
length day 11	**temp**	**1**	**1.11E5**	**15.31**	**.0002**		
	**pH**	**1**	**1.09E5**	**15.11**	**.0003**		
	**food**	**2**	**8.28E4**	**11.43**	**.0001**	low = switch < high	
	**temp × pH**	**1**	**6.66E4**	**9.20**	**.0046**	*Interaction*	*Interaction*
	temp × food	2	1.31E3	0.18	.837	8.0 (26 > 30)	26 (8.0 < 7.6)
	pH × food	2	2.38E3	0.33	.722	7.6 (26 = 30)	30 (8.0 = 7.6)
	temp × pH × food	2	5.70E3	0.79	.455		
	residual	54	7.24E3				
width day 11	**temp**	**1**	**5.38E4**	**17.69**	**.0003**		
	**pH**	**1**	**3.73E4**	**12.27**	**.0007**		
	**food**	**2**	**2.67E4**	**8.76**	**.0005**	low = switch < high	
	**temp × pH**	**1**	**2.27E4**	**7.46**	**.0085**	*Interaction*	*Interaction*
	temp × food	2	315.26	0.10	.903	8.0 (26 > 30)	26 (8.0 < 7.6)
	pH × food	2	1.10E3	0.36	.700	7.6 (26 = 30)	30 (8.0 = 7.6)
	temp × pH × food	2	3.76E3	1.24	.293		
	residual	54	3.04E4				

length day 18	**temp**	**1**	**5.85E4**	**12.10**	**.0021**	26 > 30		
	**pH**	**1**	**3.85E4**	**7.96**	**.0088**	8.0 > 7.6		
	**food**	**2**	**9.07E4**	**18.77**	**.0001**	low < switch < high		
	temp × pH	1	8.58E3	1.76	.199			
	temp × food	2	1.21E4	2.51	.095			
	pH × food	2	1.24E3	0.26	.776			
	residual	32	4.83E3					
width day 18	**temp**	**1**	**3.20E4**	**15.19**	**.0006**	26 > 30		
	**pH**	**1**	**1.16E4**	**5.30**	**.0274**	8.0 > 7.6		
	**food**	**2**	**3.87E4**	**18.37**	**.0001**	low < switch < high		
	temp × pH	1	3.23E3	1.53	.222			
	temp × food	2	4.86E3	2.31	.121			
	pH × food	2	594.66	0.28	.754			
	residual	32	2.11E3					
% abnormal	temp	1	2.99E3	22.46	.0001			
day 11	pH	1	290.25	2.18	.146			
	food	2	367.41	2.76	.070			
	temp × pH	1	47.80	0.36	.563			
	**temp × food**	**2**	**524.82**	**3.94**	**.0225**	*Interaction*	*Interaction*	
	pH × food	2	18.81	0.14	.873	low, switch (26 < 30)	26 (low = switch = high)	
	temp × pH × food	2	214.08	1.61	.210	high (26 = 30)	30 (low = switch > switch = high)	
	residual	54	133.13					
% abnormal	**temp**	**1**	**4.11E3**	**6.84**	**.0119**	*Interaction*	*Interaction*	*Interaction*
day 18	**pH**	**1**	**1.73E4**	**28.78**	**.0001**	8.0; low, switch, high (26 = 30)	26; low (8.0 = 7.6)	26; 8.0, 7.6 (low = switch = high)
	food	2	316.29	0.53	.604	7.6; low (26 = 30)	26; switch, high (8.0 < 7.6)	30; 8.0 (low = switch < high)
	**temp × pH**	**1**	**5.68E3**	**9.47**	**.0045**	7.6; switch, high (26 > 30)	30; low (8.0 < 7.6)	30; 7.6 (low > switch = high)
	temp × food	2	981.64	1.64	.204		30; switch, high (8.0 = 7.6)	
	pH × food	2	394.84	0.66	.533			
	**temp × pH × food**	**2**	**3.97E3**	**6.61**	**.0028**			
	residual	47	600.07					
Early brachiolaria	**temp**	**1**	**0.15**	**5.68**	**.0228**	26 > 30		
day 11	**pH**	**1**	**0.18**	**6.77**	**.0136**	8.0 > 7.6		
	**food**	**2**	**0.25**	**9.30**	**.0006**	low = switch < high		
	temp × pH	1	3.99E-2	1.49	.230			
	temp × food	2	6.56E-4	0.02	.976			
	pH × food	2	4.80E-2	1.79	.182			
	temp × pH × food	2	6.06E-3	0.23	.797			
	residual	44	2.68E-2					
Late brachiolaria	**temp**	**1**	**0.60**	**42.13**	**.0001**	*Interaction*	*Interaction*	
day 18	**food**	**2**	**7.11E-2**	**4.95**	**.0217**	low, switch, high (26 > 30)	26 (low = switch < switch = high)	
	**temp × food**	**2**	**5.37E-2**	**3.74**	**.0479**		30 (low = switch = high)	
	residual	17	1.44E-2					

Supplementary Table 2. Outcomes of ANOVA analyses examining the effects of two temperatures (26, 30 °C), two pH levels (pH 8.0, 7.6), and three food treatments (low, switch, high) on the length:width ratio of *Acanthaster* sp. larvae at 11 and 18 dpf (days post-fertilisation) in a flow-through seawater system. Larvae were fed *Proteomonas sulcata* three times per day at a rate equivalent to 1 × 10^3 cells mL^{-1} (low), 1 × 10^3 cells mL^{-1} from 3–11 dpf and 5 × 10^4 cells mL^{-1} thereafter (switch), or 5 × 10^4 cells mL^{-1} (high). df, degrees of freedom; MS, mean square; temp, temperature. Significant factors are in bold (p < .05).

Parameters	Source	df	MS	F	*p*	*Post hoc*
day 11	temp	1	1.30E-4	0.03	.852	
	**pH**	**1**	**0.03**	**6.87**	**.0120**	**8.1 > 7.6**
	**food**	**2**	**2.78E-2**	**7.39**	**.0020**	**low = switch < high**
	temp × pH	1	0.01	3.47	.072	
	temp × food	2	2.59E-3	0.69	.460	
	pH × food	2	2.13E-3	0.57	.574	
	temp × pH × food	2	1.48E-3	0.39	.673	
	residual	54	3.76E-3			
day 18	temp	1	4.08E-3	1.44	.239	
	**pH**	**1**	**0.02**	**8.49**	**.0069**	**8.1 > 7.6**
	food	2	6.07E-3	2.14	.142	
	temp × pH	1	1.33E-5	4.69E-3	.947	
	temp × food	2	5.21E-3	1.84	.181	
	pH × food	2	5.11E-3	1.80	.187	
	residual	32	2.84E-3			

Supplementary Table 3. Seawater conditions for the experiment examining the effects of temperature, pH, and food treatment on size, development, and survival of *Acanthaster* sp. larvae: Mean salinity, temperature (°C), pH_T (total scale), partial pressure of dissolved carbon dioxide (pCO_2), calcite saturation state (ΩCa), bicarbonate (HCO_3^-), and carbonate (CO_3^{2-}) concentrations of seawater in 12 treatments. Values for pCO_2, HCO_3^-, CO_3^{2-}, and ΩCa were calculated using values measured for salinity, pH_T, temperature, and total alkalinity (mean 2168.8 µmol kg^{-1} ± 63.4 SD, *n* = 41) in CO2SYS[1]. Values in parentheses are standard deviations, *n* = 14–38.

Treatment			Salinity	Temperature	pH_T	pCO_2	HCO_3^-	CO_3^{2-}	ΩCa
Food treatment	Temperature	pH		°C		µatm	µmol kg^{-1}	µmol kg^{-1}	
Low	26	8.0	34.6 (1.2)	26.0 (0.4)	8.03 (0.03)	390.8 (30.2)	1682.3 (48.2)	202.8 (10.9)	4.9 (0.2)
	26	7.6	34.3 (0.7)	26.6 (0.6)	7.63 (0.01)	1134.9 (42.7)	1944.3 (41.3)	94.6 (3.3)	2.3 (0.1)
	30	8.0	35.0 (1.2)	30.2 (0.4)	8.03 (0.02)	386.7 (29.2)	1621.6 (43.8)	226.9 (11.5)	5.5 (0.2)
	30	7.6	34.9 (1.2)	29.7 (0.5)	7.60 (0.03)	1225.1 (108.6)	1922.6 (57.7)	99.1 (8.8)	2.4 (0.2)
Switch	26	8.0	34.6 (0.9)	26.0 (0.4)	8.04 (0.02)	378.9 (25.7)	1671.3 (44.0)	205.8 (8.3)	5.0 (0.2)
	26	7.6	34.7 (1.2)	26.7 (0.7)	7.63 (0.03)	1165.5 (91.7)	1953.3 (49.1)	94.6 (7.0)	2.3 (0.2)
	30	8.0	35.0 (1.2)	30.5 (0.7)	8.02 (0.03)	393.6 (29.8)	1610.7 (50.2)	223.2 (12.2)	5.4 (0.3)
	30	7.6	34.9 (1.2)	29.9 (0.3)	7.61 (0.03)	1197.5 (93.1)	1917.3 (57.0)	101.3 (7.0)	2.5 (0.2)
High	26	8.0	34.3 (0.7)	26.1 (0.3)	8.04 (0.02)	382.7 (21.0)	1674.6 (42.1)	203.7 (7.8)	4.9 (0.2)
	26	7.6	34.3 (0.7)	26.4 (0.5)	7.64 (0.02)	1122.4 (63.5)	1942.9 (46.1)	94.8 (3.6)	2.3 (0.1)
	30	8.0	35.0 (1.2)	30.5 (0.7)	8.02 (0.03)	392.1 (30.2)	1610.1 (50.6)	223.5 (12.1)	5.4 (0.3)
	30	7.6	35.0 (1.2)	30.0 (0.3)	7.60 (0.03)	1233.3 (85.6)	1922.2 (55.4)	99.3 (7.1)	2.4 (0.2)

Supplementary References:

1. Pierrot, D., Lewis, E. & Wallace, D.W.R. MS Excel Program Developed for CO_2 System Calculations. Carbon Dioxide Information Analysis Center, U.S. Department of Energy, Oak Ridge National Laboratory, Oak Ridge, Tennessee; 10.3334/CDIAC/otg.CO3332SYS_XLS_CDIAC3105a (2006).
