# Supplementary material for: Variable food alters responses of larval crown-of-thorns starfish to ocean warming but not acidification
Source: Commun Biol. 2023 Jun 14;6:639. doi: 10.1038/s42003-023-05028-1 (PMC10267210; doi:10.1038/s42003-023-05028-1)
Supplement: Supplementary file 3 — Reporting Summary [file 42003_2023_5028_MOESM3_ESM.pdf]

nature portfolio

Corresponding author(s): Benjamin Mos

Last updated by author(s): May 24, 2023

Reporting Summary

Nature Portfolio wishes to improve the reproducibility of the work that we publish. This form provides structure for consistency and transparency in reporting. For further information on Nature Portfolio policies, see our Editorial Policies and the Editorial Policy Checklist.

Statistics

For all statistical analyses, confirm that the following items are present in the figure legend, table legend, main text, or Methods section.

n/a	Confirmed	
☐	☒	The exact sample size (*n*) for each experimental group/condition, given as a discrete number and unit of measurement
☐	☒	A statement on whether measurements were taken from distinct samples or whether the same sample was measured repeatedly
☐	☒	The statistical test(s) used AND whether they are one- or two-sided<br>*Only common tests should be described solely by name; describe more complex techniques in the Methods section.*
☐	☒	A description of all covariates tested
☐	☒	A description of any assumptions or corrections, such as tests of normality and adjustment for multiple comparisons
☐	☒	A full description of the statistical parameters including central tendency (e.g. means) or other basic estimates (e.g. regression coefficient) AND variation (e.g. standard deviation) or associated estimates of uncertainty (e.g. confidence intervals)
☐	☒	For null hypothesis testing, the test statistic (e.g. *F*, *t*, *r*) with confidence intervals, effect sizes, degrees of freedom and *P* value noted<br>*Give P values as exact values whenever suitable.*
☒	☐	For Bayesian analysis, information on the choice of priors and Markov chain Monte Carlo settings
☐	☒	For hierarchical and complex designs, identification of the appropriate level for tests and full reporting of outcomes
☐	☒	Estimates of effect sizes (e.g. Cohen's *d*, Pearson's *r*), indicating how they were calculated

Our web collection on statistics for biologists contains articles on many of the points above.

Software and code

Policy information about availability of computer code

Data collection	Larvae were photographed using a digital camera mounted on the dissecting microscope (MIchrome 20). Length and width of all larvae were measured from photographs using freely-available ImageJ image analysis software.
Data analysis	Data were analysed using commercially available statistical software packages, Primer (v6) and IBM SPSS (v27).

For manuscripts utilizing custom algorithms or software that are central to the research but not yet described in published literature, software must be made available to editors and reviewers. We strongly encourage code deposition in a community repository (e.g. GitHub). See the Nature Portfolio guidelines for submitting code & software for further information.

Data

Policy information about availability of data

All manuscripts must include a data availability statement. This statement should provide the following information, where applicable:

- Accession codes, unique identifiers, or web links for publicly available datasets
- A description of any restrictions on data availability
- For clinical datasets or third party data, please ensure that the statement adheres to our policy

The datasets supporting this article are available free to download from the Dryad Digital Repository: https://doi.org/10.5061/dryad.msbcc2g2f. All other data is available from the corresponding author on reasonable request.

Human research participants

Policy information about studies involving human research participants and Sex and Gender in Research.

Reporting on sex and gender	N/A
Population characteristics	N/A
Recruitment	N/A
Ethics oversight	N/A

Note that full information on the approval of the study protocol must also be provided in the manuscript.

Field-specific reporting

Please select the one below that is the best fit for your research. If you are not sure, read the appropriate sections before making your selection.

☐ Life sciences ☐ Behavioural & social sciences ☒ Ecological, evolutionary & environmental sciences

For a reference copy of the document with all sections, see nature.com/documents/nr-reporting-summary-flat.pdf

Ecological, evolutionary & environmental sciences study design

All studies must disclose on these points even when the disclosure is negative.

Study description	Experimental treatments consisted of two temperatures (26, 30 *C), two pH (pH 8.0, 7.6) and three food levels ('low': 1000 cells per mL, 'switch': 1000 cells per mL from 3-11 dpf (days post-fertilisation), followed by 50000 cells per mL thereafter, and 'high': 50000 cells per mL) factorially crossed, creating a total of 12 treatments. There were seven replicates for each treatment.
Research sample	Groups (typically 20 individuals) of Acanthaster sp. embryos and larvae (from fertilisation until they were ready to settle) taken from replicates. The mean from each replicate was used as the data to maintain independence among replicates and avoid pseudo-replication due to the potential for larvae to influence the response of other larvae in the same replicate (e.g. density effects).
Sampling strategy	Sample sizes are consistent with or exceed standard practice for ocean acidification/ocean warming research on larvae.
Data collection	Data were collected and recorded using standard procedures by one researcher, and independently verified by at least one other researcher.
Timing and spatial scale	Data were collected between February and April, 2021. Data on water quality was collected most days, and data on larval morphology, development, and survival were collected at regular time points (e.g. weekly, every 3 days). The data were collected within a laboratory experiment conducted at the National Marine Science Centre in Coffs Harbour, Australia
Data exclusions	For morphological measurements, replicates with fewer than 9 larvae were excluded from further analysis. All other data was included.
Reproducibility	This experiment is similar in design and set-up to previous studies by our lab and other research groups that have tested the effects of ocean acidification and ocean warming on Crown-of-thorns starfish (CoTS), and separate experiments by our lab and other research groups testing the effects of food availability on CoTS larvae. Our study is the first to test all three factors together. The response of the larvae in our experiment are similar to the results of previous experiments.
Randomization	Embryos/larvae were haphazardly allocated to replicates. Replicates were randomly assigned to treatments.
Blinding	Blinding was not performed. Survival, morphological, and developmental measurements were made using standard procedures performed by one researcher, and independently verified by at least one other researcher.

Did the study involve field work? ☐ Yes ☒ No

Reporting for specific materials, systems and methods

We require information from authors about some types of materials, experimental systems and methods used in many studies. Here, indicate whether each material, system or method listed is relevant to your study. If you are not sure if a list item applies to your research, read the appropriate section before selecting a response.

Materials & experimental systems

n/a	Involved in the study
☒	☐ Antibodies
☒	☐ Eukaryotic cell lines
☒	☐ Palaeontology and archaeology
☐	☒ Animals and other organisms
☒	☐ Clinical data
☒	☐ Dual use research of concern

Methods

n/a	Involved in the study
☒	☐ ChIP-seq
☒	☐ Flow cytometry
☒	☐ MRI-based neuroimaging

Animals and other research organisms

Policy information about studies involving animals; ARRIVE guidelines recommended for reporting animal research, and Sex and Gender in Research

Laboratory animals	Acanthaster sp. larvae, aged 0-60 days.
Wild animals	Adult Acanthaster sp. were collected from the Great Barrier Reef near Cairns, Queensland as part of a 'pest' removal program and transported via air freight to Coffs Harbour, Australia. Gametes were collected from four females and four males using standard protocols. Adults were maintained in captivity until they died or were donated to a public aquarium.
Reporting on sex	Data were collected from groups of larvae (presumably) comprised of both sexes. It is not practical to determine the sex of larvae without killing them.
Field-collected samples	The study did not involve samples collected from the field.
Ethics oversight	No ethical approval was required because the study did not involve a vertebrate or cephalopod invertebrate.

Note that full information on the approval of the study protocol must also be provided in the manuscript.
